# Pilot prospective open-label one-arm trial investigating intrathecal Adenosine in neuropathic pain after lumbar discectomy

**DOI:** 10.1186/s13104-020-05133-y

**Published:** 2020-06-12

**Authors:** Dawood Aghamohammadi, Mahmood Eydi, Alireza Pishgahi, Azam Esmaeilinejad, Neda Dolatkhah

**Affiliations:** 1 Palliative Care Medicine Department, Faculty of Medicine, Tabriz University of Medical Sciences, Tabriz, Iran; 2 Physical Medicine and Rehabilitation Research Center, Aging Research Institute, Tabriz University of Medical Science, Tabriz, Iran; 3 Physical Medicine and Rehabilitation Department, Faculty of Medicine, Tabriz University of Medical Sciences, Tabriz, Iran

**Keywords:** Adenosine, Intrathecal injection, Discectomy, Neuropathic pain

## Abstract

**Objectives:**

Adenosine has an analgesic and anti-inflammatory role and its injections are used for perioperative pain management. We aimed to study efficacy of intrathecal injection of adenosine for post-operative radicular pain after lumbar discectomy. Forty patients with unilevel lumbar discectomy who had radicular lower limb pain were treated by 1000 micrograms of intrathecal injection of adenosine in this single-arm prospective open-label trial between November 2015 to October 2016. Radicular pain severity using visual analogue scale (VAS) and pain killer consumption per day were assessed during a 3 months follow up period.

**Results:**

Radicular pain severity was significantly reduced in 3 month follow-up period in comparison to the baseline (F = 19,760, df = 2.53, p-value < 0.001). Further, painkiller medication consumption rate in average during 3 month follow-up period after injection was significantly lower in comparison to baseline (F = 19.244, df = 1.98, p-value < 0.001). This study suggests that intrathecal injection of 1000 micrograms adenosine is a safe and effective method for post-operative neuropathic pain management after uni-level disk surgeries.

*Trial registration* IRCT201608171772N20, Retrospectively registered on 2016-08-28.

## Introduction

Neuropathic pain is a multifaceted pain feature, showing an inconvenient procedure of regular sensory signaling changed so that pain is sensed in the nonexistence of a nociceptive motivation, or replies to ordinarily deleterious stimuli are heightened. The management of persistent postoperative neuropathic pain (PPNP) has preoccupied a multi-discipline approach comprising pharmacological, interventional, and surgical proceeding [1, 2].

Adenosine which has four subtypes of A1, A2a, A2b and A3, is an endogenous protein nucleotide. It modulates physiologic processes that play role in nociception, anti-nociception and central and peripheral analgesia. Its plasma level increases during cell injury, inflammation and ischemic events in order to prevent cell damage from the ischemic-hypoxic reactions [3]. Adenosine injection can reduce perioperative pain through activation of A1 receptor by anti-nociceptive or analgesic mechanism [4, 5].

Intravenous injection of adenosine in the surgical procedures has reduced analgesic requirement by 20–50% in studies. It also limits systolic blood pressure fluctuations caused by painful stimuli during a surgery. Studies also have reported a 18–26% reduction of postoperative opioid consumption in the breast and uterine surgical procedures [3]. Intrathecal injection of 500–2000 micrograms of adenosine has been associated with spinal hyperalgesia and allodynia [6].

Since studies regarding postoperative analgesic effects of adenosine are not sufficient to overcome controversy in the literature, in this study, we aimed to determine the efficacy of intrathecal adenosine injection for relief of neuropathic pain after lumbar uni-level discectom.

## Main text

### Methods

#### Participants

This is a single-arm prospective open-label trial investigating intrathecal adenosine injection for relief of the neuropathic pain after lumbar uni-level discectomy in 40 patients with history of uni-level lumbar discectomy in the last 3–6 months and chief complaint of postoperative radicular neuropathic pain which was conducted at the Emam Reza Educational Hospital, Tabriz, Iran. Recruitment started in November 2015 and lasted until October 2016. Inclusion criteria were age between 20 and 70 years old; recent history of lumbar uni-level discectomy and history of postoperative radicular pain. Those patients with a history of asthma; liver and renal disease; endocrine and coronary disease; sensitivity to xanthine like dipyridamole and aminophylline; maldigestion of methyl-xanthine; addiction; cardiac arrhythmia and psychogenic disorders were excluded. Recruited patients were seen at 3 visits: initial visit (visit 1), 1-month visit (visit 2), and 3-month visit (visit 3), and their data were collected in a dedicated case report form. This study adhered to the CONSORT guidelines.

#### Measurements

Demographic characteristics of patients and the level of surgery were recorded at the beginning. Before study intervention, radicular pain severity using visual analogue scale (VAS) was assessed. It is a 10 number numerical scale in which “1” indicates *No Pain* and “10” was equal to *Most Severe Pain*. Finally amount of painkillers consumption per day (number of tablets per day) was recorded.

In first 48 h after injection with 2 h interval and at the end of 1st, 2nd, 3rd, 4th, 8th and 12th weeks post injection, severity of radicular pain and amount of painkillers per day were recorded. At 1st, 2nd and 3rd month post injection, headache severity and sleep quality were also evaluated. All the measurements and side effects after discharge from hospital were recorded by telephone calls.

#### Intrathecal injection

Under sterile condition and cardiac monitoring, a secure intravenous line for hydration by normal saline was established while patient was seated relaxes. Interlaminar space between 4th (L4) and 5th (L5) lumbar vertebras which is in same level with posterior superior iliac spine protuberances was marked. A 25 gauge spinal needle was used to fenestrate L4–L5 interlaminar space in the midline. Needle was pushed slowly to the intrathecal space. As soon as cerebrospinal fluid was observed in the syringe hub, 1000 micrograms of adenosine were injected to the intrathecal space. The entrance site was bandaged and patient was transferred to recovery unit. If there was no reports of sensorimotor or autonomic dysfunction, patient was transmitted to pain unit in order to be observed for 48 h.

#### Statistics analysis

Data were analyzed by SPSS (V.21, IBM, New York, New York, USA). Statistical analyses comprised a report of patient characteristics descriptive analyses. Repeated measures of ANOVA and Chi square tests were also recruited. In this study, p-values less than 0.05 were considered as significant.

### Results

Forty patients with history of radicular neuropathic pain after lumbar unilevel surgery were included in this trial from November 2015 to October 2016. Mean age of participants was 50.55 ± 10.53 years with a male: female ratio of 21:19. The baseline radicular pain and pain killer consumption were 6.62 ± 0.70 and 4.62 ± 0.70, respectively. Eight patients had unilevel lumbar discectomy plus vertebral fusion. It means that based on surgeon decision they had a more complex condition and needed more comprehensive management. Six patients (15 percent) had done heavy work after the block.

**Fig. 1:**
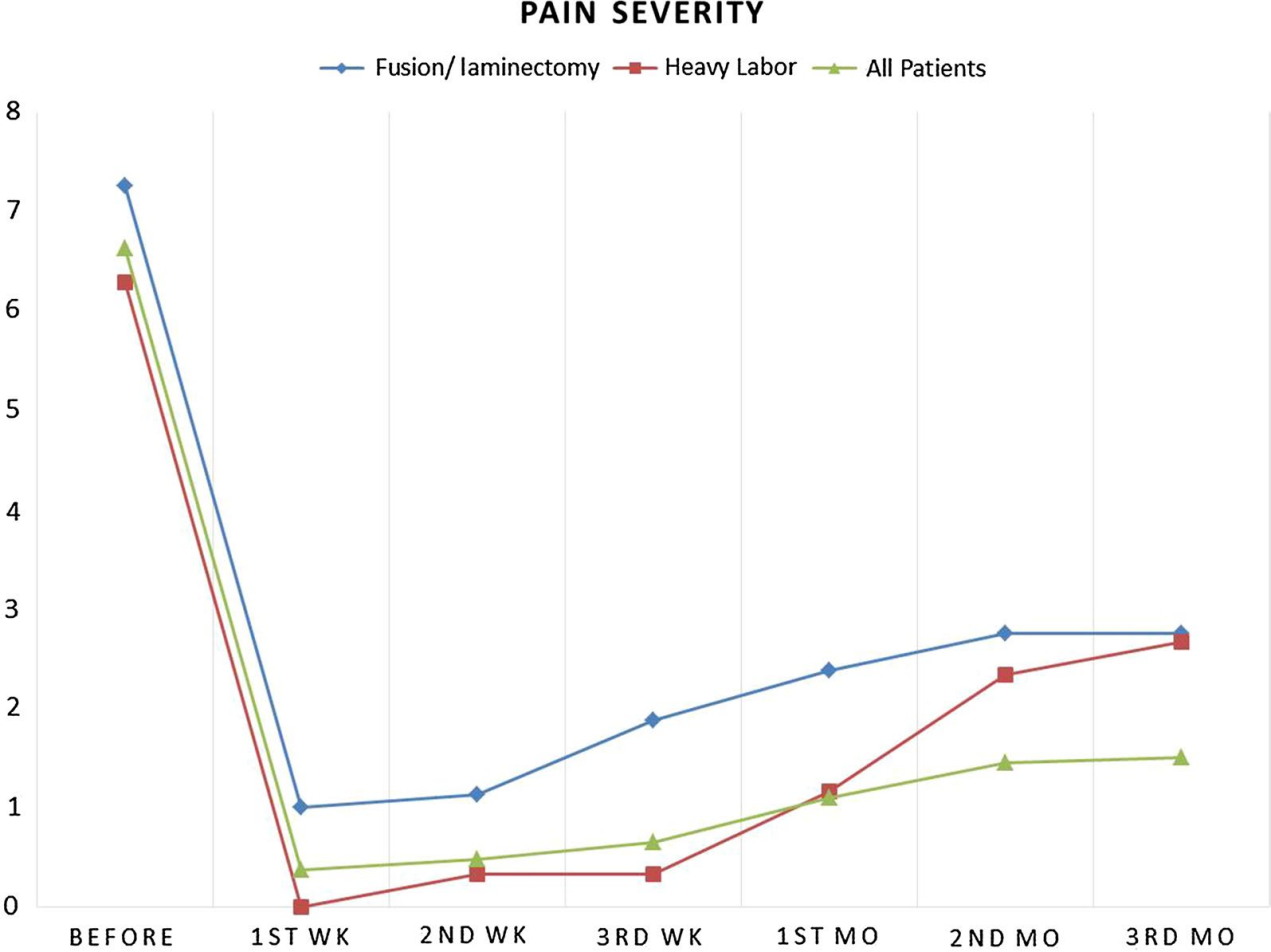
Comparison of pain severity between three groups of study

Table 1 shows pain severity and pain killer consumption in all participants, participants with heavy labor and participants with complex surgery groups. Radicular pain severity was significantly reduced in first week follow-up period in comparison to the baseline (6.62 ± 0.70 to 0.37 ± 1.00, p < 0.001). Although pain intensity has an ascending pattern from week 1 to month 3 post injection follow up (0.37 ± 1.00 to 1.52 ± 1.61, p = 0.041) but it was still lower than baseline measurements (F = 19,760, df = 2.53, p-value < 0.001) (Fig. 1).

**Table 1:** Pain severity and Pain killer consumption in all participants, participants with heavy labor and participants with complex surgery groups

	Pain severity (VAS)						Pain killer consumption per day					
	1 wk	2 wk	3 wk	1 m	2 m	3 m	1 wk	2 wk	3 wk	1 m	2 m	3 m
All participants (n = 40)												
Mean	0.37	0.47	0.65	1.1	1.45	1.52	0.22	0.17	0.32	0.7	0.9	0.95
SD	1	1.08	1.31	1.51	1.63	1.61	0.53	0.44	0.69	0.91	0.032	0.06
Heavy labor experience (n = 6)												
Mean	0	0.33	0.33	1.16	2.33	2.66	0	0.16	0.16	0.83	1.66	2
SD	0	0.81	0.81	1.47	1.21	0.51	0	0.4	0.4	0.98	0.81	0.63
Laminectomy and CD replacement (n = 8)												
Mean	1	1.12	1.87	2.37	2.75	2.75	0.87	0.5	1	1.5	1.75	1.87
SD	1.06	0.99	0.99	0.74	0.88	0.88	0.64	0.53	0.75	0.53	0.7	0.83

*SD* standard deviation, *hr* hour, *wk* week, *mo* mo

**Fig. 2:**
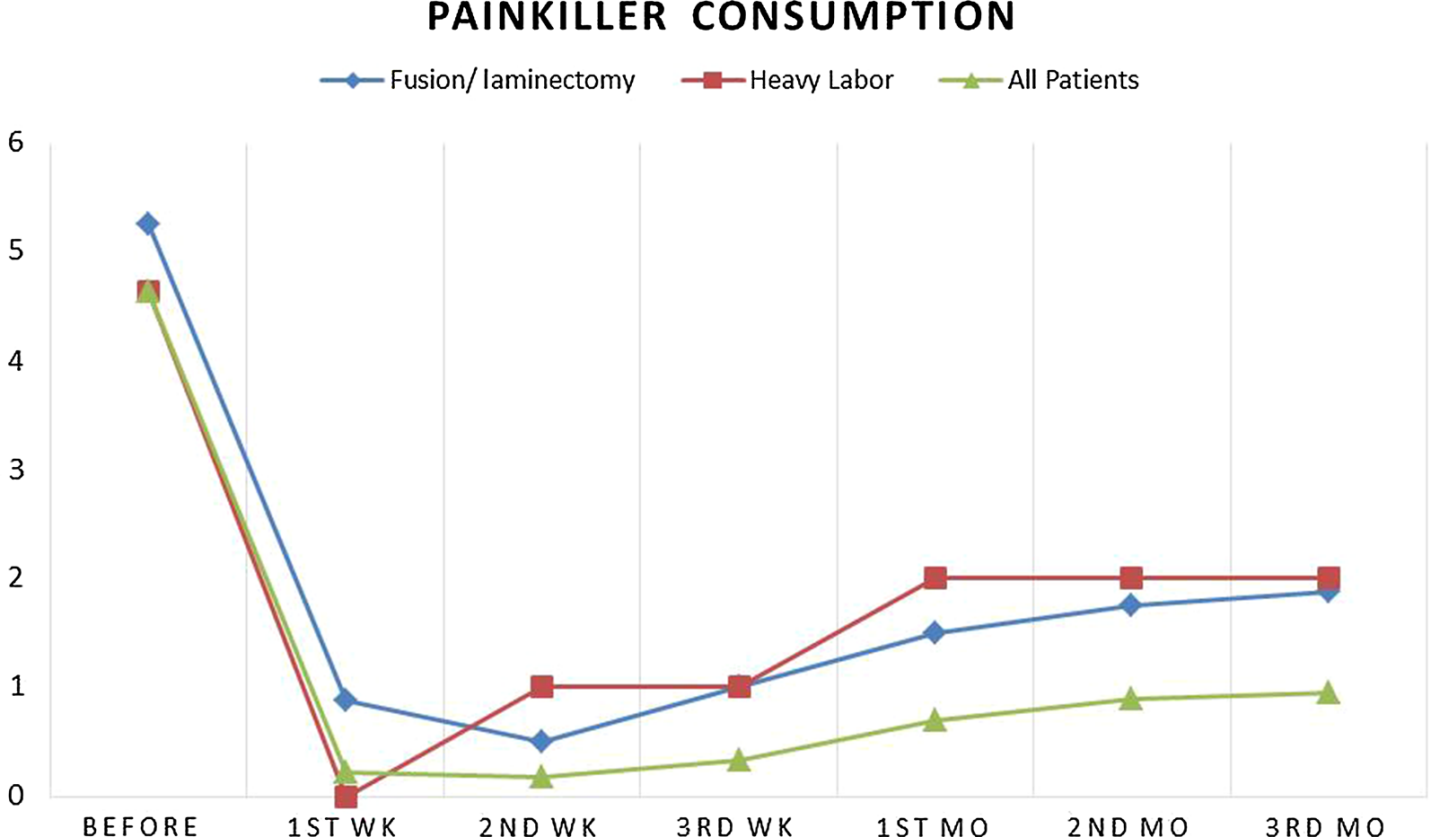
Comparison of painkiller consumption between three groups of study

Painkiller consumption was significantly reduced in first week follow-up period in comparison to the baseline (4.62 ± 0.70 to 0.22 ± 0.53, p < 0.001). Although painkiller consumption has an ascending pattern from week 1 to month 3 post injection follow up (0.22 ± 0.53 to 0.95 ± 0.06, p = 0.121) but it was still lower than baseline measurements (F = 19.244, df = 1.98, p-value < 0.001) (Fig. 2). In this study none of the patients consumed painkillers in first 48 h after injection.

In 3 months follow up period after intrathecal injection of adenosine, patients with unilevel lumbar discectomy plus vertebral fusion had higher level of pain severity. Interestingly those patients who had experienced heavy labor duties in follow up period after injection (n = 6) also had higher level of mean radicular pain in comparison to other patients (Fig. 1). Both of these groups had also consumed more painkillers (Fig. 2).

Fortunately during follow-up period of this clinical trial there were no reports of significant complications due to intrathecal adenosine injection. Intrathecal adenosine didn’t affect blood pressure, heart rate and motor or sensory testing. Additionally, there was no report of postspinal headache or superficial wound infections. However, 10 of participants had transient pain localized to the lumbar region at the time of injection for less than 30 min.

### Discussion

In this study, forty patients with history of unilateral or bilateral radicular pain after lumbar disc unilevel surgery were treated by 1000 micrograms of adenosine intrathecal injection. In the 3 month period of follow up, pain severity and painkiller consumption rate per day were significantly reduced and fortunately no serious complications were reported. James et al. [7] had similar findings. In their study intrathecal adenosine injection had led to reduction of neuropathic pain. However they could not find any difference between 500 Âµg and 2000 micrograms of adenosine injection in case of pain relief, but higher doses were associated with more complications. Rauck et al. [8] also suggested that adenosine in adjunction with clonidine may reduce neuropathic pain sensations.

Adenosine as an endogenous porin nucleotide has four subtypes of A1, A2a, a2b and A3. It is elevated in blood plasma during cell inflammatory injury in order to prevent hypoxic-ischemic cell damage. Adenosine receptors mechanism of action is based on G-protein complex. A1 receptor links to G i/o protein and reduces cyclic adenosine monophosphate (cAMP) level. A2 works through Gs protein and promotes adenylate cyclase action. A1 also can bind to Go and inhibits calcium transduction. A2b and A3 bind to Gq and stimulate phospholipase action. So A2a subtype is responsible for anti-inflammatory effects of adenosine. In summary, A1 receptor has analgesic and anti-nociceptive effects while A2a and A3 promote anti-inflammatory mechanisms [1–3]. There is an alternative theory that believes in nociceptive effects of A1 agonists in spinal and cortical levels through *N*-methyl-d-aspartate transmission [9, 10].

Adenosine and Remifentanil analgesic effect for post-operative pain reduction were evaluated in Fukunga et al. study. Pain severity score was 60% lower in adenosine group in comparison to the remifentanil one. Also opioid consumption rate of adenosine treated patients were significantly lower in comparison to the remifentanil treated patients. However, they noted higher blood levels of carbon-dioxide, systolic blood pressure disturbance and higher heart rates in adenosine treated group [11].

Also there were no evidence of complications in current study but clinical trials in literature have reports of headache, back and lower limbs pain [7, 11]. These side effects have been seen in those patients who had received doses of adenosine above 2000 micrograms per injection. Adenosine dose of 1000 Âµg may be the reason for lack of complication in current study.

Surprisingly, Yamaoka et al. and Sharma et al. who had tried intrathecal injections of adenosine for post-operative pain management could not find any significant analgesic effect [12, 13]. Yamaoka states that reduction of A1 receptor in postoperative period is responsible for these findings. In current study, those patients who had experienced heavy labor duties (n = 6) after injection and those who had more complex surgeries, combination of laminectomy and Vertebral fusion (n = 8) experienced less analgesic effects.

### Conclusion

This study suggests that intrathecal injection of adenosine is a safe and effective method for post-operative pain management after uni-level disk surgeries.

## Limitations

There are limitations for this study. Firstly, this study did not have a control group. The most important reason for this limitation was lack of eligible cases based on inclusion criteria, but it would be much better if analgesic effects of adenosine have been compared with a control therapy. Secondly, measuring pain killer consumption per day based on number of pills reported by the patient through telephone calls is not completely accurate but since patients were not able to count the milligram of drugs they had consumed, authors had forced to simplify this measurement, for patients of study. Finally an important confounding factor is daily job which could directly impact pain sensation severity reported by the patient. Although authors of current study have recorded heavy labor duties in follow up period but it would be better if results have been classified based on daily job heaviness.

## Data Availability

All the necessary data are presented herewith. However if needed, raw data on excel format can be availed on reasonable request from the corresponding author.

## Abbreviations

cAMP: Cyclic adenosine monophosphate
SD: Standard deviation
VAS: Visual analogue scale
